# Supplementary material for: Assembly reactions of SARS-CoV-2 nucleocapsid protein with nucleic acid
Source: bioRxiv. 2023 Nov 23:2023.11.22.568361. Preprint. [Version 1] doi: 10.1101/2023.11.22.568361 (PMC10690241; doi:10.1101/2023.11.22.568361)
Supplement: Supplement 1 [file media-1.pdf]

Supplementary Figure S2

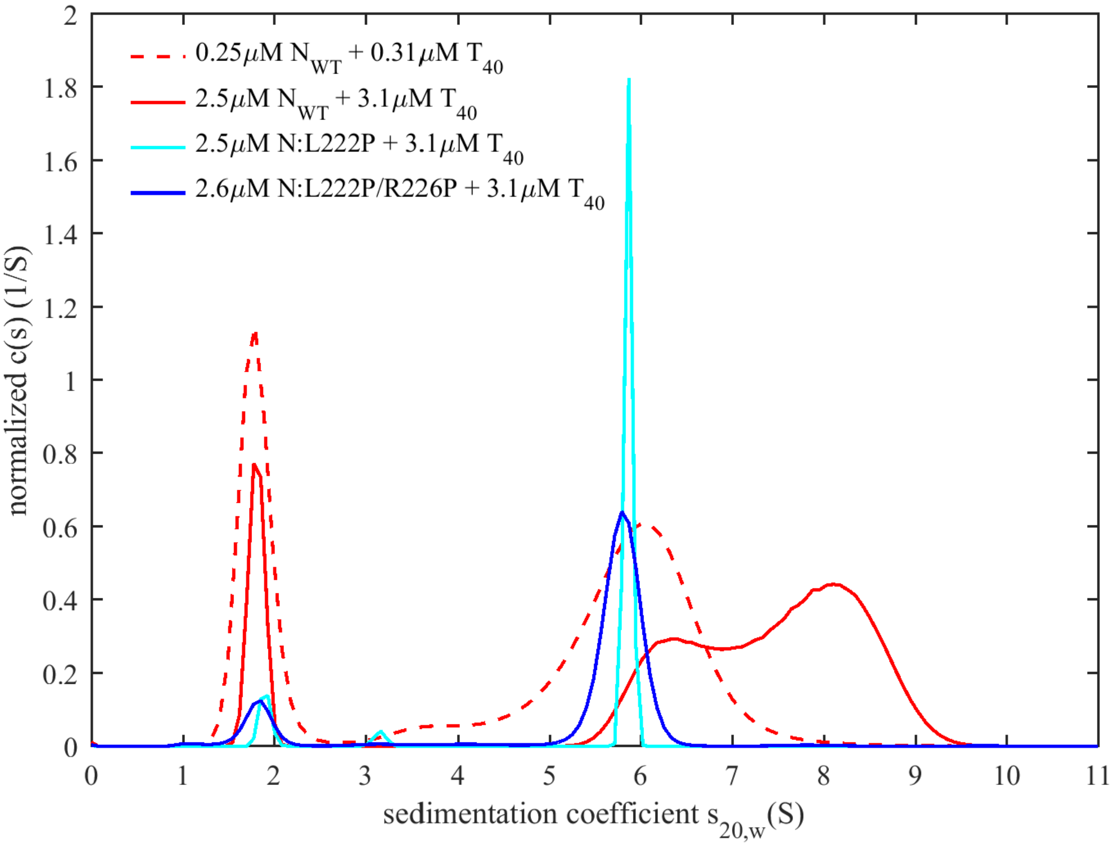

Supplementary Figure S2: Simultaneous LRS oligomerization and scaffolding on the oligonucleotide T_{40} in moderate ionic strength buffer B_{65K}. Shown are sedimentation coefficient distributions recorded at 260 nm for N_{WT} (red) and the LRS mutants N:L222P (cyan, magnitude reduced by factor 5) and N:L222P/R226P (blue, magnitude reduced by factor 2) at concentrations indicated.
