# Supplementary material for: Assembly reactions of SARS-CoV-2 nucleocapsid protein with nucleic acid
Source: bioRxiv. 2023 Nov 23:2023.11.22.568361. Preprint. [Version 1] doi: 10.1101/2023.11.22.568361 (PMC10690241; doi:10.1101/2023.11.22.568361)
Supplement: Supplement 2 [file media-2.pdf]

Supplementary Figure S3

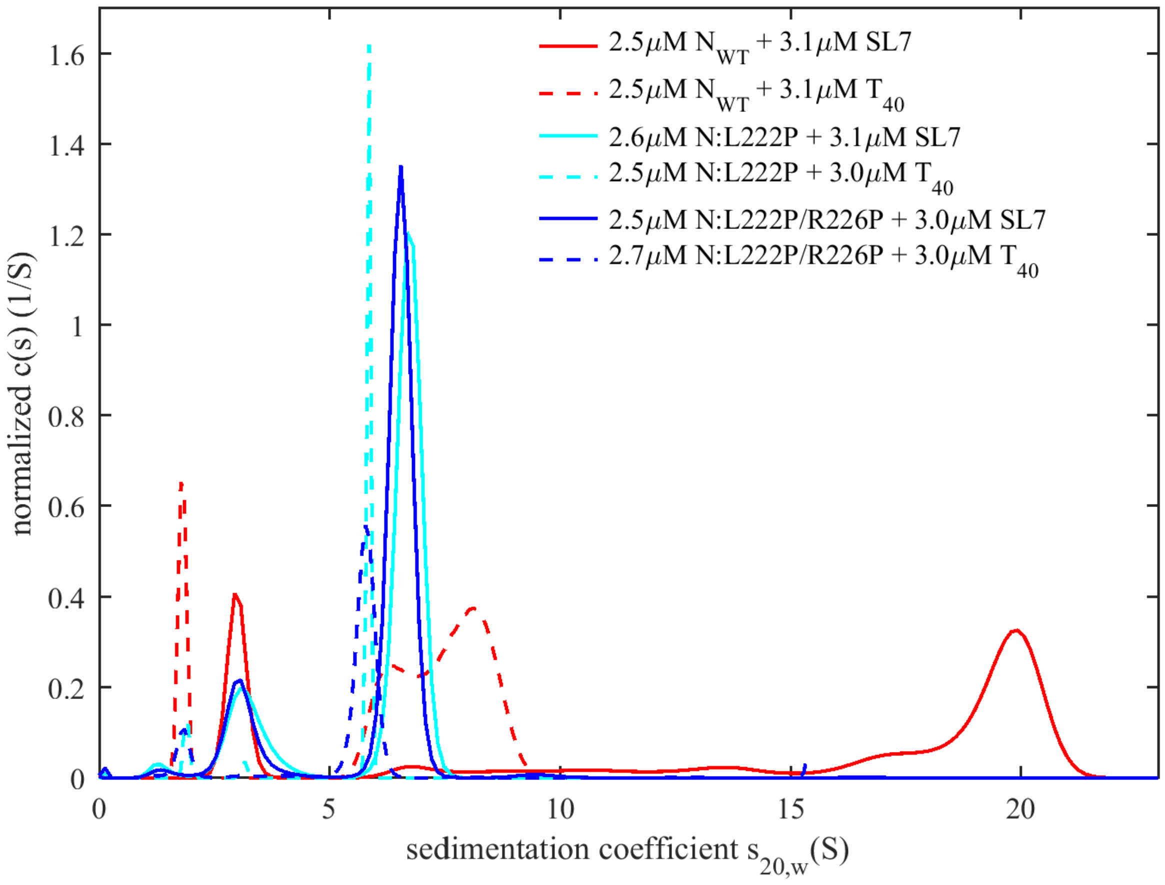

Supplementary Figure S3: Comparison of RNP formation with oligonucleotide T_{40} and stem-loop SL7. Shown are sedimentation coefficient distributions recorded at 260 nm for T_{40} (dashed lines) and SL7 (solid lines) with N_{WT} (red) and the LRS mutants N:L222P (cyan) and N:L222P/R226P (blue) all at the same concentrations. Buffer conditions are at moderate ionic strength, identical to those of the RNP assay in MP (B_{65K}). This figure reproduces the data from **Supplementary Figure S2** and **Figure 5** to enable a direct visual comparison.
