# Supplementary material for: Assembly reactions of SARS-CoV-2 nucleocapsid protein with nucleic acid
Source: bioRxiv. 2023 Nov 23:2023.11.22.568361. Preprint. [Version 1] doi: 10.1101/2023.11.22.568361 (PMC10690241; doi:10.1101/2023.11.22.568361)
Supplement: Supplement 3 [file media-3.pdf]

Supplementary Figure S4

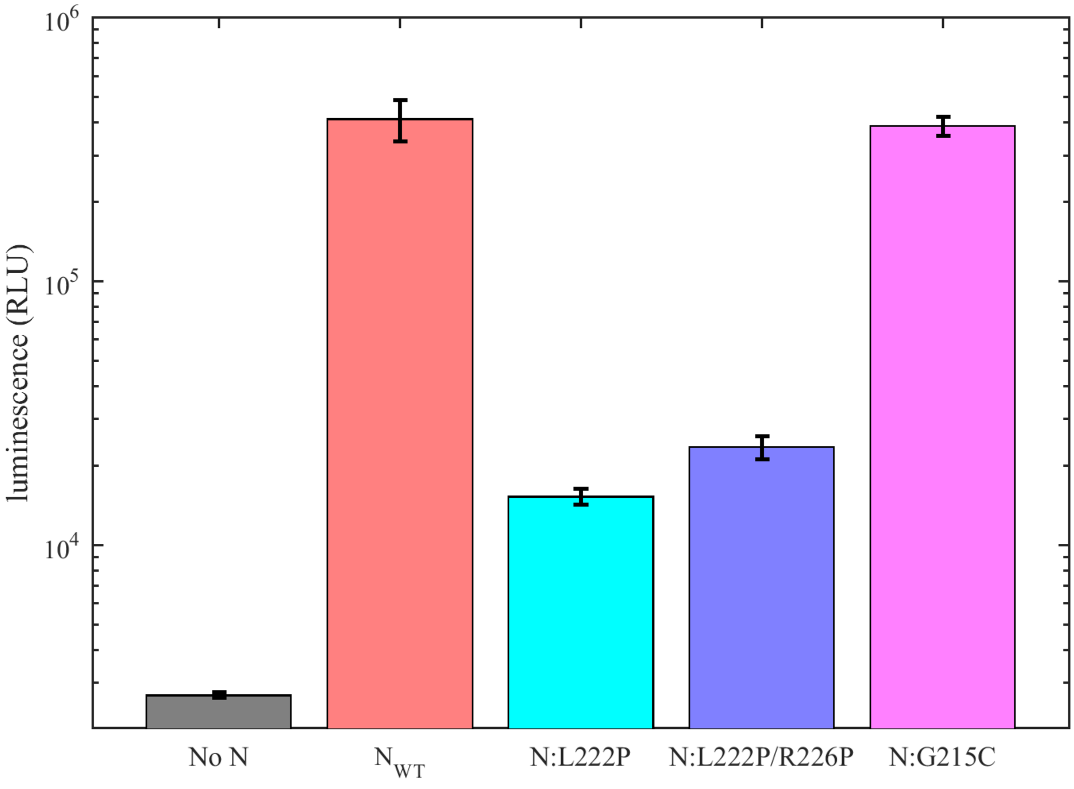

Supplemental Figure S4: VLP assembly in the presence of GSK3 inhibitor CHIR98014. Luminescence after incubation of VLP producer cells with 1.25 µM CHIR98014 inhibiting phosphorylation, thereby reducing non-assembly competent populations of intracellular N-protein. The efficiency of VLP formation was measured for different (or lacking) N-protein species indicated and quantified in relative luminescence units.
