# Supplementary material for: Assembly reactions of SARS-CoV-2 nucleocapsid protein with nucleic acid
Source: bioRxiv. 2023 Nov 23:2023.11.22.568361. Preprint. [Version 1] doi: 10.1101/2023.11.22.568361 (PMC10690241; doi:10.1101/2023.11.22.568361)
Supplement: Supplement 5 [file media-5.pdf]

Supplementary Figure S1

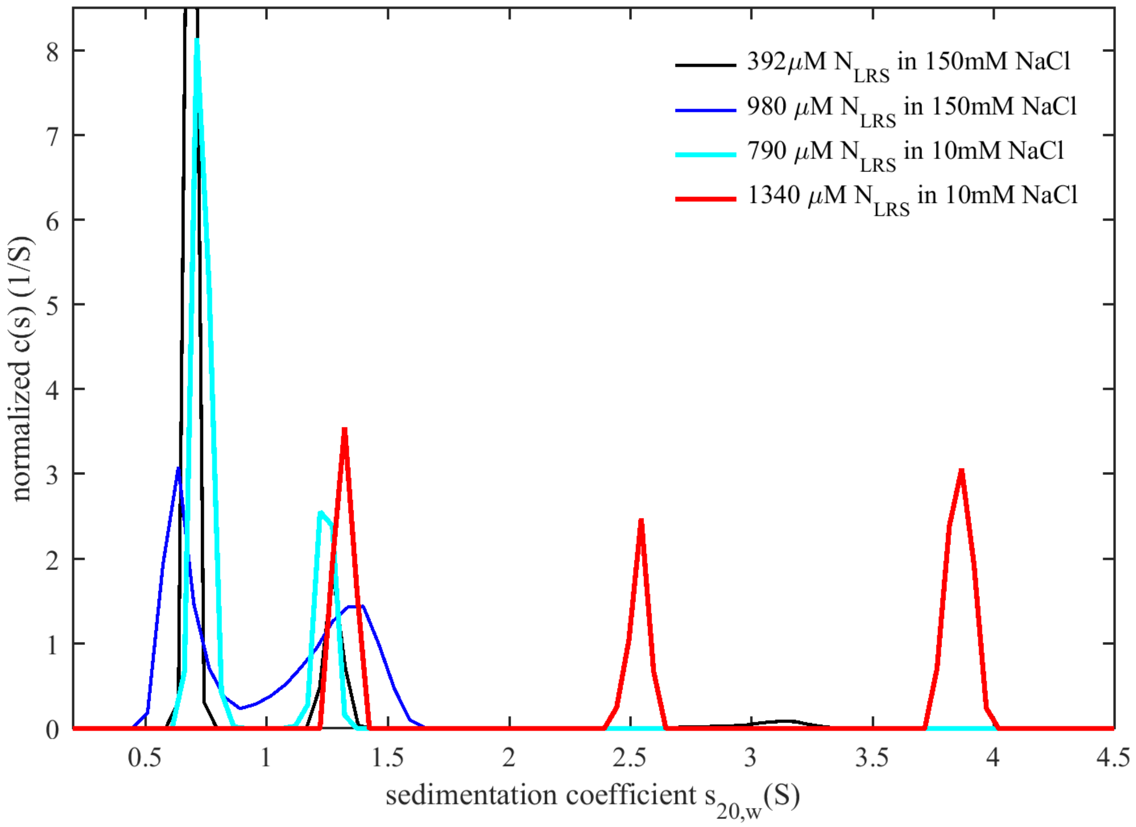

Supplementary Figure S1: Enhancement of protein-protein interaction in the LRS at low ionic strength. Shown are sedimentation coefficient distributions recorded for the peptide N:210-246 comprising the LRS transient helix in either phosphate buffered saline with 150 mM NaCl (black and blue curves) or in buffer B_{10Na} containing 10 mM NaCl (cyan and red curves).
